# SHAPING COVID-19 INTERVENTIONS THAT ADDRESS SOCIAL BARRIERS AMONG OLDER LATINOS IN ASSISTED LIVING FACILITIES

**DOI:** 10.1093/geroni/igad104.3590

**Published:** 2023-12-21

**Authors:** Isis Panellas, Richard Beaulaurier

**Affiliations:** Florida International University, Miami, Florida, United States; Florida International University, Miami, Florida, United States

## Abstract

COVID-19 has exposed severe gaps in our understanding of preventing infectious diseases, particularly in congregate living facilities. While the medical and public health communities developed clear insights into preventing or limiting the spread of infection, our understanding of barriers to adopting these measures has lagged, particularly in minority communities. The COVID-19 pandemic has disproportionately affected older Latino adults, particularly those living in assisted living facilities. Among this vulnerable population, older Latinos face unique social barriers such as language and communication difficulties, lack of culturally competent care, and socioeconomic disadvantages that contribute to their increased susceptibility to COVID-19 transmission and which can lead to more severe outcomes. While initial intervention strategies focused on medical interventions to mitigate the virus’s effects, there has been a gradual recognition that barriers to prevention have increasingly shifted from medical to social domains. This paper aims to document the existing knowledge and identify knowledge gaps concerning effectively managing these social barriers. Culturally sensitive approaches must consider salient features of Latino family dynamics such as allocentrism, familismo, respeto, and expected gender roles. In addition, there are often barriers related to external factors, such as a lack of Spanish language resources, community engagement, stereotypes, and racism. Prevention approaches need to identify and address these barriers to safeguard the well-being of older Latinos in assisted living facilities during the COVID-19 pandemic. This review will explore our current understanding of these social barriers, provide insights into potential strategies to mitigate their impact and identify continuing gaps in our knowledge.

